# Segmentation of Older Adults in the Acceptance of Social Networking Sites Using Machine Learning

**DOI:** 10.3389/fpsyg.2021.705715

**Published:** 2021-08-11

**Authors:** Patricio E. Ramírez-Correa, F. Javier Rondán-Cataluña, Jorge Arenas-Gaitán, Elizabeth E. Grandón, Jorge L. Alfaro-Pérez, Muriel Ramírez-Santana

**Affiliations:** ^1^School of Engineering, Universidad Católica del Norte, Coquimbo, Chile; ^2^Department of Business Administration and Marketing, University of Seville, Seville, Spain; ^3^Department of Information Systems, University of Bío-Bío, Concepción, Chile; ^4^Department of Public Health, Universidad Católica del Norte, Coquimbo, Chile

**Keywords:** acceptance, elderly, social network sites, heterogeneity, machine learning

## Abstract

This study analyzes the most important predictors of acceptance of social network sites in a sample of Chilean elder people (over 60). We employ a novelty procedure to explore this phenomenon. This procedure performs apriori segmentation based on gender and generation. It then applies the deep learning technique to identify the predictors (performance expectancy, effort expectancy, altruism, telepresence, social identity, facilitating conditions, hedonic motivation, perceived physical condition, social norms, habit, and trust) by segments. The predictor variables were taken from the literature on the use of social network sites, and an empirical study was carried out by quota sampling with a sample size of 395 older people. The results show different predictors of social network sites considering all the samples, baby boomer (born between 1947 and 1966) males and females, silent (born between 1927 and 1946) males and females. The high heterogeneity among older people is confirmed; this means that dealing with older adults as a uniform set of users of social network sites is a mistake. This study demonstrates that the four segments behave differently, and many diverse variables influence the acceptance of social network sites.

## Introduction

Research on the use that older people make of social network site (SNS) is growing, although they are few compared to other age groups. One of the first works was performed by Arjan et al. (2008), who studied the difference in the relationships of built-in SNS as a function of age. They observed the greater age diversity among the contacts that the elderly have, including younger relatives who act as prescribers for the SNSs use. Subsequently, Ji et al. (2010) proposed identifying older users' patterns and older non-users of SNSs and their differences in use. Curran and Lennon (2013) examined the influence of sociological variables, such as social influence and social tension, on the intention to use SNSs among older adults. In a similar vein, Peral-Peral et al. (2015) verified differences among the elderly depending on the tools considered, utilitarian or hedonic, such as a SNS. The differences they found are due more to psychographic than to demographic factors. Psychographic characteristics include lifestyle and group identity of individuals (Mirvis and Kanter, 1991; Gilbert and Warren, 1995), such as interests, activities, opinions, values, and attitudes; this type of variables have been vastly used as segmentation bases (Lin, 2002; Bakir, 2020). On the other hand, characteristics of users based on demographics include gender differences, age, ethnic background, income, occupation, education, household size, religion, generation, nationality, and even social class.

In general, the ranges of years for each generation have differences among countries. For example, Karashchuk et al. (2020) explained that the range of years for each generation has specific differences between Russia and America. Accordingly, in the case of the Baby Boomer generation, the Baby Boomer birth period varies from country to country (Sudbury-Riley et al., 2015). Moreover, although some studies propose the search for heterogeneity within generational cohorts (Rajaobelina et al., 2021), other studies have indicated that the differences are not associated with demographic variables such as age but rather psychographic variables such as cognitive age (Arenas-Gaitán et al., 2020). In this context, we follow the recent study of Ramírez-Correa et al. (2019a) that operationalized the Chilean generations in a digital setting.

To understand consumer behavior as a user of SNSs, age is a concept that may be too simple (Chaney et al., 2017). The idea of generation is much broader and more valuable since it implies the shared experience of a society and a set of events that influence the shared values that are at the base of their behaviors (Strauss and Howe, 1997). It is generally accepted a period of 20 years to define a generation (Chaney et al., 2017). The Silent Generation is a generation that lived through the First and Second World War and the Great Depression. These events favored that people in this generation appreciate discipline, hard work, authority, loyalty, and self-denial and overall are socially and financially conservative. They distrust change and prefer the status quo, and a majority worry is outliving their assets. Therefore, they are inclined to save money and continue saving money now (Li et al., 2013).

On the other hand, Baby Boomer generation lived through the movement for the fight for Civil Rights, the liberalization of women, and the arrival of men on the moon. Since these events, with increased educational, financial, and social opportunities, the Baby Boomer generation is often portrayed as a generation of optimism, exploration, and achievement. In addition, they are the first generation to grow up with television, and this technology strongly influenced them. They value individual choice, community involvement, prosperity, ownership, self-actualising, and wellness (Li et al., 2013).

We can show these differences in two examples of the scientific literature. First, Silent generation is regarded as being unconditionally loyal to the employer and the organization; instead, Baby Boom generation is only loyal to a certain extent; they tend to view the organization as the driving force behind their careers (Valickas and Jakštaite, 2017). Second, Silent generation and Baby Boomer differed in trip motivation and trip activities. In North America, the boomers were more like younger generations and looked for more energetic experiences like physical excitement, adventure, and time away from home with their families. Meanwhile, Silent generation behaved more traditionally and pursued more static experiences such as casinos, cooking, history, and culture (Lehto et al., 2008).

All these differences among generations may be the basis of behaviors and use different from those of the SNSs. Previous studies had reported differences in age and gender among SNSs older adult users. In terms of age, it has been found that the Baby Boomer generation is more likely than the Silent generation to use social media. For example, in a study conducted by Pew Research Center (2017), 45% of boomers say they ever use social networking sites, compared with 20% of silent. Regarding gender, it has been found that older women outperform older men in their use of different types of SNSs (Pew Research Center, 2013). In particular, in 2014, the Pew Report Center informed that 52% of online older women, compared to 39% of older men, use social media. Furthermore, concerning the use of Facebook, Randall et al. (2015) reported that boomer women used this SNS in a more significant proportion than their counterpart silent generation.

Another stream of research has focused on finding motivations and barriers for the elderly using SNSs. For instance, the work of Jung and Sundar (2016) focused on the motivations and barriers encountered by the elderly in the use of Facebook. They found that the main reasons for using this SNS are staying connected, sharing pictures, social surveillance, responding to family and friends, and satisfying nosiness. On the other hand, the main obstacles facing older adults are privacy, the need to spend in the media and the unfamiliarity with technology. In addition, and considering that there are multiple SNSs today, Rondán-Cataluña et al. (2020) found essential differences in the demographic and psychographic characteristics among the greatest users of Facebook, YouTube, Instagram, Twitter, and WhatsApp.

There is a body of research (e.g., Pesonen et al., 2015; Villarejo-Ramos et al., 2019) that shows a high heterogeneity concerning the use of new technologies by the elderly. In the specific case of SNSs, Villarejo-Ramos et al. (2019) identified five segments of elders with respect to the employment of two kinds of services: the use of SNSs as a hedonic service (these services are mainly related to entertaining, enjoyment, feelings and pleasure) and electronic banking as a utilitarian service (Niemelä-Nyrhinen, 2007; Arenas-Gaitán et al., 2020). Villarejo-Ramos et al. found essential differences among segments depending on the elderly's demographic and psychographic characteristics.

Technology acceptance is a widely established topic in the information systems literature. The term “technology acceptance” refers to the level of willingness an individual possesses in adopting a technology (Dillon, 2001). Within this context, models like the Technology Acceptance Model (TAM) proposed by Davis (1986), the Unified Theory of Acceptance and Use of Technology (UTAUT) proposed by Venkatesh et al. (2003) or UTAUT2 proposed by Venkatesh et al. (2012) have been the most frequently used research frames (Rondan-Cataluña et al., 2015). Compared to previous models, UTAUT2 provides a more aligned view of consumer technologies. Previous models were developed for mandatory technologies like those used in the workplace. In addition, to the variables contained in the UTAUT2 model, other psychographic variables have been incorporated. In this sense, Peral-Peral et al. (2015) showed that these types of variables had a high explanatory power of SNSs in older adults. Moreover, UTAUT2 has generally been used as a segmentation analysis tool (Arenas-Gaitán et al., 2020).

Most of these models have been applied using structural equation models (SEM) such as LISREL, AMOS, or PLS-PM as statistical tools. The use of these tools implies assuming linear relationships among the variables that make up these models. Nonetheless, research has questioned the linearity of relationships in the models of technology adoption (Rondan-Cataluña et al., 2015). However, the most recent works that have applied non-linearity to technology acceptance models have used the Artificial Neural Networks (ANN) analysis tool (Sharma et al., 2016; Ooi et al., 2018; Chavoshi and Hamidi, 2019; Kalinić et al., 2019).

This study aims to analyze the most important predictors of SNSs use in a sample of Chilean elder people (over 60). To this end, we first analyze the factors that determine the level of SNSs acceptance by expanding with psychographic variables the extended UTAUT2. Then, considering differences in the adoption of technologies depending on the elders' generation and gender (Ramírez-Correa et al., 2019b), we compare their behavior based on these variables, mainly focusing on the Baby Boomer and Silent generations.

Based on the objective mentioned above, we will answer the following research questions: (a) Could the UTAUT2 model's constructs be linked using a non-linear procedure to make good predictions of SNSs use in the elderly population? (b) Could we obtain better predictions of SNSs use through gender and generation as segmentation bases? (c) Are there different behaviors among older Chilean people concerning SNSs use?

## Method

### Methodology Framework

**Figure 1:**
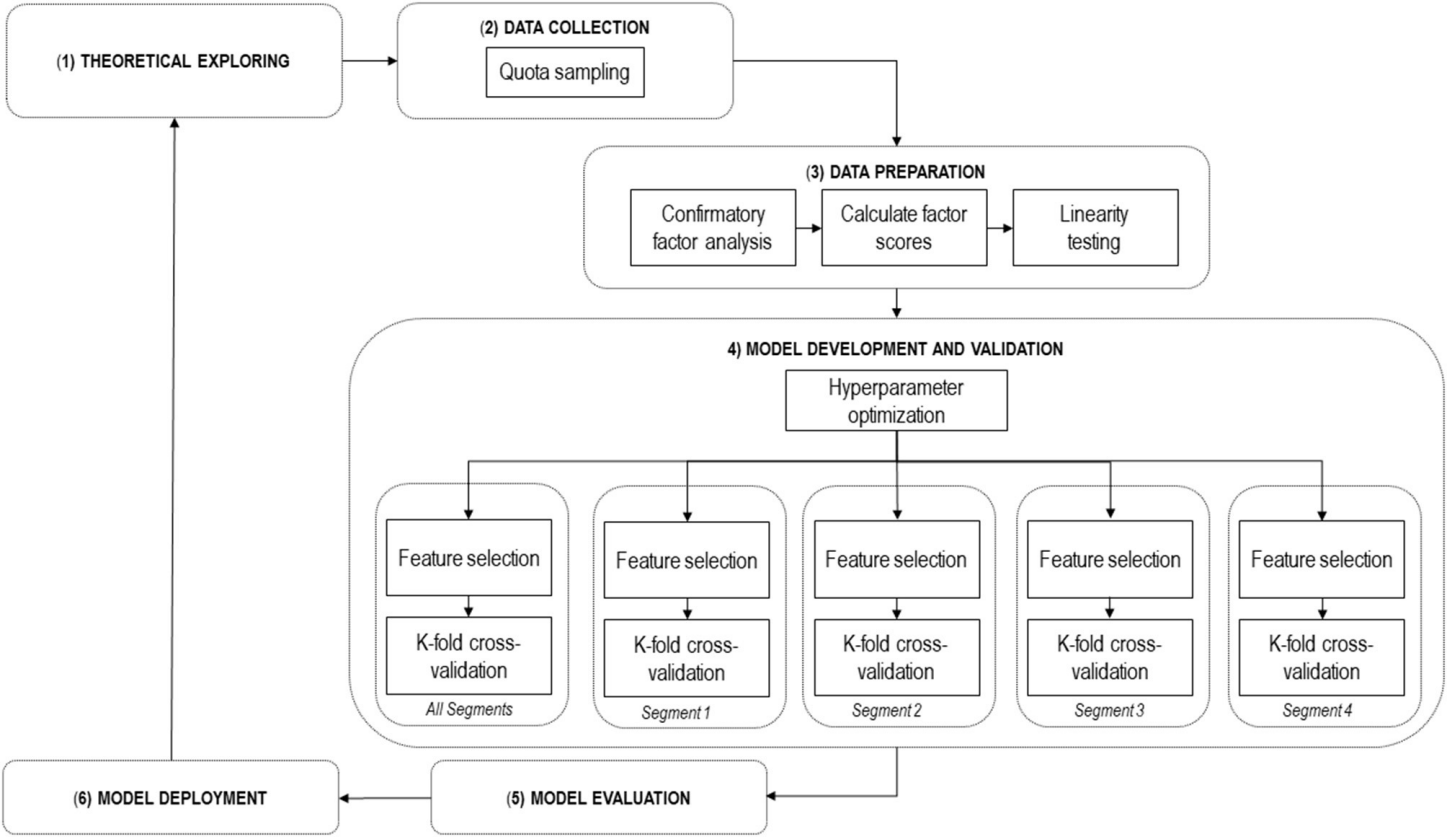
Methodology framework.

Figure 1 represents the methodological framework used in this study. The first phase, called ***Theoretical Exploring*, **consists of an exploratory analysis of the literature, whose product is a set of possible determinants of SNSs use. In this case UTAUT2 constructs were selected.

The second phase, called ***Data Collection*, **conducts surveys of SNSs users following a quota sampling. Quota sampling is a type of non-random sampling which includes identifying segments within a population, how many individuals to be included in each segment to make up the ideal sample, then choosing this sample based on convenience sampling (Hibberts et al., 2012).

The third phase, called ***Data Preparation*, **involves three steps: (1) confirming the good fit of the measurement model through a confirmatory factor analysis (Gallagher and Brown, 2013); (2) the calculation of standardized scores for each factor (Rizopoulos, 2006); (3) testing the linearity between determinants and SNSs usage (Field et al., 2012).

The fourth phase, called ***Model Development and Validation*, **is the core of the analysis. This phase is focused on using Deep Learning (DL) to predict SNSs use. DL is a sophisticated ANN that is increasingly used for problems that involve machine learning processes. A crucial advantage of an ANN concerning modeling traditional techniques is that it can capture linear and non-linear relations, not including demanding distribution assumptions, such as normality, linearity, and homoscedasticity. DL introduces plasticity into even very complex models, non-linear relationships between determinants and result variables, a condition that has enabled DL to move beyond models from traditional machine learning (Kraus et al., 2020). The use of non-linear functions and several single-layer networks are the main improvements in this procedure. The use of DL for classification and prediction purposes is a recent research line. These procedures have been used in many different applications (Liskowski and Krawiec, 2016; Sa et al., 2016; Sladojevic et al., 2016; Havaei et al., 2017; Ozturk et al., 2020). However, using this computational model to predict SNSs use by older adults is an innovative application. The use of DL for prediction purposes opens a new way of understanding behavior in social sciences.

The basic procedure for using DL involves establishing a computational model from a data set named training data and then applying it to a new data set to assess its performance level to make predictions. In this study, this phase is composed of several steps. Initially, an optimization of the model's hyperparameters is carried out. The model parameters refer to the characteristics of the training data acquired during the model's training. On the other hand, the model hyperparameters refer to the properties that regulate this whole process. Specifically, hyperparameters refer to parameters regulating the design of the model (Aggarwal, 2018). Next, the determinants of SNSs use are selected using the feature selection technique. Feature selection denotes to identifying relevant features and deleting non-relevant, noisy, or redundant data (Kumar and Minz, 2014). This procedure is carried out for the total sample as well as for each of the user segments. The final step in this phase is to apply a K-fold cross-validation procedure to each group of analyses, train a DL model, and test this model's predictive capacity. K-fold cross-validation refers to a method that obtains a set of n examples and divides them into K sets of size n/K (called folds). For every fold, one model is trained on the other K-1 folders (Langford, 2005). Additionally, this step serves to determine the importance of predictors.

The next phase, called ***Model Evaluation*, **conducts a discussion of the results of the previous phase. Finally, the phase called ***Model Deployment***is related to using the model to generate public policies on older adults using SNSs.

The first phase findings were presented in the theoretical background section. The present section describes the second phase. Next, the Results section provides the outcomes of phases three and four. Finally, the Discussion and Conclusions section provides elements associated with this methodological framework's final two phases.

### Data Collection

A questionnaire was conducted through a personal interview with older people from two cities in Chile. After filtering the questionnaires, a total of 395 valid cases remained.

We carried out a stratified sample procedure. People over 60 who reported that they had used the Internet in the last 3 months were taken into account as users. Paid surveyors conducted the fieldwork. Surveyors were carefully selected based on their background in social and medical sciences areas. In addition, surveyors have been trained to ensure they know how to approach older adults and understand survey concepts. Due to the stratified sampling requirements, an online record system was used as a daily mechanism to control the whole data collection process. Due to the process explained below, all the questionaries were verified and validated.

We segment the sample of the elderly population into these two different generational cohorts, the Silent and Baby Boomers generations. The Silent generation is composed of people born between 1927 and 1946 who witnessed events such as the great depression, the growth of Nazism, and World War II. On the other hand, a baby boomer is born between 1947 and 1966; it is the generation of the seventies who observed the hippie era, the cold war, and the political polarization (Ramírez-Correa et al., 2019a). We have also considered the gender variable because, in the elderly, society expects different purposes and has educated men and women differently. Gender differences in value priorities varied considerably among the baby boomers (Lyons et al., 2005) and between men and women travelers of the Silent generation (Pennington-Gray and Lane, 2002).

Stratified sampling was conducted because the nature of the study precluded the analysis of all elements of the population. Some specifics of this procedure are described below. The cities of Coquimbo and Concepción were considered as they have a larger share of the population over the age of 60 in Chile. Based on data from the National Statistical Institute of Chile, the proportion of older adults in Coquimbo is 16.7%. In comparison, in Concepción the figure is 17.3%. As a sampling frame, the most recent population census data available in the country for the cities of Coquimbo and Concepción were used. Since it has become important to categorize people by generation to explain their behavior on the Internet, generation has been used as the stratification variable. The IX Survey of Internet Access and Use of the Undersecretary of Telecommunications of Chile was used to determine the proportion of Internet users by gender and generation; this information constitutes the latest data available in the country. After filtering this database and determining the proportions, a total of 156,401 older adults were identified. Because the many variables to be collected and given that there is no information about the variance of each stratum, a stratified procedure by simple affixation to determine the sample size was planned, considering for this a maximum error allowed of 5%. The procedure is set out below. First, the total sample size is calculated using the random sampling formula for a finite sample. For the total sample, a confidence level of 95% is also taken. Hence, the calculation of the global sample indicated a size of 395. Second, the global sample is divided into the strata by simple affixation. The distribution of the stratified sample is the following: 281 users belong to the Baby Boomer generation (41 men and 49 women in Coquimbo, 84 men and 107 women in Concepción), and 114 users belong to the Silent generation (17 men and 22 women in Coquimbo, 31 men and 44 women in Concepción); in total 173 men and 222 women.

The study was carried out following the Helsinki Declaration and approved by the local Ethics Committee of the Universidad Católica del Norte (R14/2019).

All the scales have been adapted from earlier studies in the context of SNSs use (see Table 1). The original scales were made in English, and other studies have validated these scales translated into Spanish, either for general or elderly information technology users (Arenas-Gaitán et al., 2011; Arenas-Gaitan et al., 2013; Arenas-Gaitán et al., 2019; Ramírez-Correa et al., 2019b). We measured the constructs using a 7-point Likert scale ranging from “strongly disagree” to “strongly agree.” The measurements for actual use, performance expectancy, effort expectancy, altruism, telepresence, and social identity were developed based on Kwon and Wen's study (Kwon and Wen, 2010). Measurements for facilitating conditions and hedonic motivation were adapted from Venkatesh et al. (2012). The perceived physical condition was drawn from Ryu et al. (2009). The scales for social norms and trust were based on Sun et al. (2014). Additionally, the measurement for the habit construct was developed from Mouakket's study (Mouakket, 2015). The survey is available in the Appendix.

**Table 1:** References of scales measurement.

**Latent variable**	**Number of items**	**References**
Actual Use (AU)	3	Kwon and Wen, 2010
Performance Expectancy (PE)	4	Kwon and Wen, 2010
Effort Expectancy (EE)	3	Kwon and Wen, 2010
Social Norms (SN)	4	Sun et al., 2014
Facilitating Conditions (FC)	3	Venkatesh et al., 2012
Hedonic Motivation (HM)	3	Venkatesh et al., 2012
Habit (HA)	5	Mouakket, 2015
Trust (TR)	3	Sun et al., 2014
Telepresence (TE)	4	Kwon and Wen, 2010
Social Identity (SI)	3	Kwon and Wen, 2010
Altruism (AL)	3	Kwon and Wen, 2010
Perceived Physical Condition (PPC)	3	Ryu et al., 2009

## Results

### Confirmatory Factor Analysis

Confirmatory factor analysis was conducted using the R language to verify that the data were consistent with the measurement model. This analysis indicates a satisfactory fit of the data to the measurement scales. The Comparative Fit Index (CFI) and Tucker Fit Index (TFI) values are 0.92 and 0.91, respectively. Moreover, the value of the Root Mean Square Error of Approximation (RMSEA) is 0.05. The reliability indicator of the scale associated with each latent variable ranged between 0.73 and 0.92.

### Standardized Scores and Linearity Test

We calculate standardized scores for each factor using the loading values, and then we test the linearity of the relationships between these scores and the SNSs usage score. According to the *p*-values of the linearity deviation tests, non-linear relationships were found for all the relationships studied.

### Model Development and Validation

In this study, DL models are developed using the RapidMiner Studio version 9.6 software. In particular, the analysis utilized the H2O DL algorithm. This algorithm is established on a multilayered ANN that is trained with stochastic gradient descent applying back-propagation. The algorithm was set up with the minimum number of hidden layers (with 50 neurons each), considering not losing predictive capacity. Ten iterations of the dataset were created. All the models were tested with a ten-fold cross-validation procedure. This cross-validation technique consists of two subprocesses. The first subprocess trains the model and the second subprocess applies this model. The sample is split into ten subsets of identical size, one of these subsets is retained as the test dataset, and the rest of the subsets are utilized as the training data set. The operation is replicated ten times, and each subset is used exactly once to test the model. The results from these replicates are merged into a single estimate. R^2^ was the main criterion to assess the predictive accuracy of the models.

Given the computational configuration explained above, and to determine which variables best predict the use of SNSs, we have followed the next steps.

#### Hyperparameter Optimization

An activation function of a node is a critical hyperparameter in DL and defines the output of that node given a set of inputs. The exponential rectifier linear unit function was determined by evaluating different activation functions because it provides the model's best predictive capacity. For this optimization process, the total sample and all the predictor variables initially proposed for the study were included.

#### Feature Selection

The best set of predictors was selected through an optimization process by testing all possible combinations of predictor selections. This process was executed for the entire sample and each subsample. For these processes, all the predictor variables initially proposed for the study were included.

Two steps were previously performed for the two, three, and four hidden layers configurations (with 50 neurons in each layer). Since the differences in R^2^ between the configurations is close to zero, and a configuration with more layers requires more computation time, the configuration with fewer layers was selected.

#### Cross-Validation and Determining the Importance of Predictors

Prediction processes were run for the entire sample and each subsample, taken as the selected set of predictors for each dataset. This operation established the importance of the predictors in each analysis group. Table 2 indicates the analysis findings.

**Table 2:** Predictors of SNS use.

**Dependent variable: SNS Use**	**All**	**Baby boomer males**	**Baby boomer females**	**Silent males**	**Silent females**
R^2^ (Train)	0.70	0.76	0.66	0.71	0.78
R^2^ (Test)	0.69	0.72	0.62	0.84	0.79
Predictors (% importance)	EE (22.7)	HA (39.3)	FC (23.1)	SI (51.7)	TR (26.0)
	HA (21.0)	TE (33.0)	HM (20.1)	HA (48.3)	PPC (25.2)
	SN (20.2)	SN (27.8)	SN (19.8)		PE (24.6)
	PE (19.4)		PE (19.4)		HA (24.3)
	SI (16.7)		HA (17.6)		

## Comparison of Differences Between the Segments

Table 3 shows a comparison of characteristics among the models determined for each segment. We highlight two points. First, the model associated with silent males stands out for higher coefficients of determination, and less complexity, closely followed by the model related to silent females. Second, the model associated with baby boomer females presents the lowest performance.

**Table 3:** Characteristics of the models.

**Characteristic**	**All**	**Baby boomer males**	**Baby boomer females**	**Silent males**	**Silent females**
Birth year	1927–1966	1947–1966		1927–1946	
Number of predictors	5	3	5	2	4
Sample size	395	125	156	48	66
Coefficient of determination (R^2^)	0.69	0.72	0.62	0.84	0.79
Adjusted coefficient of determination (R^2^ adjusted)	0.69	0.71	0.61	0.83	0.78
Mallow's Cp	11	7	11	5	9

In order to compare the results with a multigroup analysis that assumes linear relationships, we calculated the significant differences between the segments using Partial Least Squares Multi-Group Analysis (PLS-MGA).

Before performing the PLS-MGA, both the measurement and structural models were analyzed, and the measurement invariance was evaluated. PLS techniques were developed using the SmartPLS version 3.3.3 software.

Table 4 shows the main results of the PLS and PLS-MGA procedures. The significant path coefficients indicate a lower number of the predictors by segment, highlighting that the DL procedure considers relationships regardless of whether they are associated linearly or non-linearly. In addition, the PLS-MGA analysis points out three significant differences, two differences associated with the effect of social identity (between baby boomer females and silent males, and between silent females and silent males) and one difference associated with telepresence (between baby boomer males and silent males).

**Table 4:** PLS-MGA results.

**Predictor to USE**	**Path coefficients**				**PLS-MGA (differences)**				
	**All**	**Baby boomer males**	**Baby boomer females**	**Silent males**	**Silent females**	**Baby boomer females vs. baby boomer males**	**Baby boomer females vs. silent females**	**Baby boomer females vs. silent males**	**Baby boomer males vs. silent females**	**Baby boomer males vs. silent males**	**Silent females vs. silent males**
Altruism (ALT)	−0.03	−0.03	0.01	−0.04	0.06	0.04	−0.05	0.05	−0.09	0.01	0.10
Effort expectancy (EE)	0.08	0.02	0.11	−0.06	0.06	0.09	0.05	0.17	−0.04	0.08	0.12
Facilitating conditions (FC)	0.02	0.06	0.03	−0.03	0.05	−0.03	−0.02	0.06	0.01	0.09	0.08
Habit (HA)	**0.67**	**0.67**	**0.65**	**0.87**	**0.78**	−0.02	−0.13	−0.22	−0.11	−0.20	−0.09
Hedonic motivation (HM)	0.03	−0.01	0.10	−0.11	−0.01	0.11	0.11	0.21	0.00	0.10	0.10
Performance expectancy (PE)	**0.11**	0.06	**0.15**	0.14	0.15	0.09	0.00	0.01	−0.09	−0.08	0.01
P. physical condition (PPC)	−0.05	−0.01	**−0.10**	0.06	−0.06	−0.09	−0.04	−0.16	0.05	−0.07	−0.12
Social identity (SI)	0.02	0.03	−0.09	**0.35**	−0.12	−0.12	0.03	**−0.44**	0.15	−0.32	**−0.47**
Social norms (SN)	−0.04	0.02	−0.10	0.10	−0.01	−0.12	−0.09	−0.20	0.03	−0.08	−0.11
Telepresence (TE)	0.05	**0.17**	0.07	−0.25	0.01	−0.10	0.06	0.32	0.16	**0.42**	0.26
Trust (TR)	**0.07**	0.07	0.01	0.16	0.10	−0.06	−0.09	−0.15	−0.03	−0.09	−0.06

*Bold values denote statistical significance at the p < 0.05 level*.

## Discussion and Conclusions

We develop this discussion following the research questions written in the introduction section.

We could demonstrate that using a non-linear procedure, such as a DL algorithm, we can obtain good predictions of SNSs use in the elderly population. Considering the whole sample without segmentation, the R^2^ is 0.69. Many predictors of SNSs use show particular importance (effort expectancy, habit, social influence, and performance expectancy). The results change considerably considering generation and gender variables.

According to the second research question, we obtained better predictions of SNSs use through gender and generation as segmentation bases. Based on the results, we observe different predictors of SNSs considering the whole sample, baby boomer males, baby boomer females, silent males, and silent females. Our results are consistent with the findings of Schehl et al. (2019), who found differences explained by age and gender in older Internet users. Habit is the only relevant predictor of SNSs use in all the groups. The DL approach shows that the R^2^ of the global model is less than the R^2^ of three of the four segments studied. Therefore, the application of this apriori segmentation provides a better explanation of the phenomenon of SNSs use in Chilean elder adults. Only the segment of female baby boomers exhibits an R^2^ somewhat lower than the global R^2^.

Therefore, we found different behaviors among older Chilean people concerning SNSs use. In baby boomer males, only habit, telepresence, and social norms are significant predictors of SNSs use, quite different from their counterpart females. Besides, it is the only segment where telepresence is significant. For this segment of the elderly, SNSs are a way of feeling like they were located remotely from where they currently are. Using the emotion of telepresence, the service user will be more exposed to the other's response (Kwon and Wen, 2010). The relevant predictors for baby boomer females are facilitating conditions, hedonic motivation, social norms, performance expectancy and habit. However, the explanatory power, according to the R^2^, is the lowest. Furthermore, facilitating conditions and hedonist motivation are essential predictors of SNSs use just in this segment. For this cohort of women, using SNSs is a way of getting fun and entertainment. We also observe many differences between both generations and genders. Only social identity (which is a relevant predictor only in this group) and habit are significant predictors for silent males. However, this group achieves the highest explanatory power. They like to participate in their social group, and the activities in their social group are an essential part of their life. Furthermore, we observe that silent females are significantly impacted by trust, perceived physical condition, and performance expectancy concerning SNSs use. This group is the only one where the perceived physical condition and trust are relevant predictors of the dependent variable. This group of women makes increasingly more effort to perform everyday tasks and effortlessly contact family and friends using SNSs. Also, they feel that SNSs are secure environments to share information with other people.

From an academic standpoint, our study delves into the digital divide for the elderly. Beyond the stereotype of the elderly as people far removed from technology, our results confirm a high heterogeneity of their behaviors (Villarejo-Ramos et al., 2019; Arenas-Gaitán et al., 2020), and they point out the importance of considering both demographic characteristics, especially other psychographic characteristics (Peral-Peral et al., 2015).

From a business perspective, our results indicate that it is a mistake for companies to develop strategies for the elderly considering them as a homogeneous group. The findings suggest that generation and gender are good predictors of behaviors and can be used as efficient segmentation criteria within this market. Also, this work offers the critical variables for each group to activate their behaviors in online social networks.

From a social standpoint, older people are an increasingly important part of the population, especially in western countries. To develop active aging, communication and social participation are essential elements. In an ever more digital world, the largest social forum is currently on SNSs, and the elderly should not be oblivious to this. Based on the findings unveiled in this work, government policies to bring the elderly closer to the SNSs, and to digital technologies in general, must consider the great differences that are registered within this segment.

Considering the positive effects of SNSs use on older people's health, public policies should encourage their use, but consider the heterogeneity among them. The motivations for using SNSs are quite different between female baby boomers and female silent, or between both genders in both generations. Consequently, public policies aimed at fostering older people's SNSs use should be specific for each segment, adapting them considering every group's different motivations.

The limitations of this study drive our future research. All the respondents of this study were SNS users. To investigate the motivations of why some older people do not use SNS is quite impressive. Also, comparing Chilean elder adults with the same age group in other countries (such as Japan, China, South Korea, and others) where technology is more advanced should be considered.

## Data Availability Statement

The raw data supporting the conclusions of this article will be made available by the authors, without undue reservation.

## Ethics Statement

The studies involving human participants were reviewed and approved by Ethics Committee of the Universidad Católica del Norte (Resolution 14/2019). The patients/participants provided their written informed consent to participate in this study.

## Author Contributions

PR-C and EG undertook the research. EG and MR-S collected the data. PR-C prepared the initial manuscript and performed the data analysis. FR-C and JA-G completed, revised, and finalized the manuscript. JA-P participated in preparing the manuscript. All authors contributed to the article and approved the submitted version.

## Conflict of Interest

The authors declare that the research was conducted in the absence of any commercial or financial relationships that could be construed as a potential conflict of interest.

## Publisher's Note

All claims expressed in this article are solely those of the authors and do not necessarily represent those of their affiliated organizations, or those of the publisher, the editors and the reviewers. Any product that may be evaluated in this article, or claim that may be made by its manufacturer, is not guaranteed or endorsed by the publisher.
