# Corrigendum: Segmentation of Older Adults in the Acceptance of Social Networking Sites Using Machine Learning

**DOI:** 10.3389/fpsyg.2021.765840

**Published:** 2021-09-22

**Authors:** Patricio E. Ramírez-Correa, F. Javier Rondán-Cataluña, Jorge Arenas-Gaitán, Elizabeth E. Grandón, Jorge L. Alfaro-Pérez, Muriel Ramírez-Santana

**Affiliations:** ^1^School of Engineering, Universidad Católica del Norte, Coquimbo, Chile; ^2^Department of Business Administration and Marketing, University of Seville, Seville, Spain; ^3^Department of Information Systems, University of Bío-Bío, Concepción, Chile; ^4^Department of Public Health, Universidad Católica del Norte, Coquimbo, Chile

**Keywords:** acceptance, elderly, social network sites, heterogeneity, machine learning

In the published article, there was an error in affiliation **1** and **4**. Instead of “**Catholic University of the North**,” it should be “**Universidad Católica del Norte**.”

The authors apologize for this error and state that this does not change the scientific conclusions of the article in any way. The original article has been updated.

## Publisher's Note

All claims expressed in this article are solely those of the authors and do not necessarily represent those of their affiliated organizations, or those of the publisher, the editors and the reviewers. Any product that may be evaluated in this article, or claim that may be made by its manufacturer, is not guaranteed or endorsed by the publisher.

