# Supplementary material for: Segmentation of Older Adults in the Acceptance of Social Networking Sites Using Machine Learning
Source: Front Psychol. 2021 Aug 11;12:705715. doi: 10.3389/fpsyg.2021.705715 (PMC8385199; doi:10.3389/fpsyg.2021.705715)
Supplement: Supplementary file 1 [file Data_Sheet_1.docx]

**Appendix.**

**Survey**

Actual use (USE)

USE1 I tend to use the SNS frequently.

USE2 I spend a lot of time on SNS.

USE3 I exerted myself to SNS.

Performance Expectancy (PE)

PU1 Using the SNS enables me to acquire more information or meet more people.

PU2 Using the SNS would improve my efficiency in sharing information and connecting with others.

PU3 The SNS is a useful service for communication.

PU4 The SNS is a useful service for the interaction of members.

Effort Expectancy (EE)

EE1 Learning to use the SNS is easy for me.

EE2 The process of using the SNS is clear and understandable.

EE3 I find the SNS easy to use.

Altruism (AL)

AL I tend to encourage people who are in a real crisis or need.

AL I usually help them the solution when people ask me the solution.

AL I give congratulation when people tell me good news.

Telepresence (TE)

TE1 When the SNS ends, I felt like I actually met other people.

TE2 I felt that the SNS creates a new world.

TE3 While engaged with the SNS, I felt I was in a different society.

TE4 While engaged with the SNS, the SNS world was more real or present to me compared to the ‘‘real world”.

Social Identity (SI)

SI1 As a member of the community, my position is very important to me.

SI2 As a member of the community, I am the type of person who likes to engage in my community.

SI3 Activities in my community are an important part of my life.

Facilitating Conditions (FC)

FC1 I have the resources necessary to use SNS

FC2 I have the knowledge necessary to use SNS

FC3 SNS is compatible with other technologies I use.

FC4 I can get help from others when I have difficulties using SNS.

Hedonic Motivation (HM)

HM1 Using SNS is fun.

HM2 Using SNS is enjoyable.

HM3 Using SNS is very entertaining.

Perceived Physical Condition (PPC)

PPC1 Requires me to exert more effort to perform daily activities.

PPC2 Limits the kind of activities that I can perform.

PPC3 Causes me to have difficulty in performing daily activities.

Social Influence (SI)

SI1. People who influence my behavior think that I should use SNS.

SI2. People who are important to me think that I should use SNS.

SI3. People whose opinions I value prefer me to use SNS.

SI4. People I look up to expect me to use SNS.

Trust (TR)

TR1 SNS are safe environments in which to exchange information with others.

TR2 SNS are reliable environments in which to conduct their activities.

TR3 SNS handle personal information submitted by users in a competent fashion.

Habit (HA)

HA1 I use SNS as a matter of habit.

HA2 Using SNS has become automatic to me.

HA3 Using SNS is natural to me.

HA4 I use SNS without thinking.

HA5 Using SNS has become a routine for me.
